# Assessment of cytochrome P450 3A4-mediated drug–drug interactions for ipatasertib using a fit-for-purpose physiologically based pharmacokinetic model

**DOI:** 10.1007/s00280-022-04434-2

**Published:** 2022-04-15

**Authors:** Jing Jing, Yuan Chen, Luna Musib, Jin Y. Jin, Kit Wun Kathy Cheung, Kenta Yoshida, Rucha Sane

**Affiliations:** 1 Clinical Pharmacology, Genentech, Inc., South San Francisco, CA USA; 2 Drug Metabolism and Pharmacokinetics, Genentech, Inc., South San Francisco, CA USA

**Keywords:** PBPK, Ipatasertib, Drug–drug interaction, CYP3A4

## Abstract

**Purpose:**

Ipatasertib, a potent and highly selective small-molecule inhibitor of AKT, is currently under investigation for treatment of cancer. Ipatasertib is a substrate and a time-dependent inhibitor of CYP3A4. It exhibits non-linear pharmacokinetics at subclinical doses in the clinical dose escalation study. To assess the DDI risk of ipatasertib at the intended clinical dose of 400 mg with CYP3A4 inhibitors, inducers, and substrates, a fit-for-purpose physiologically based pharmacokinetic (PBPK) model of ipatasertib was developed.

**Methods:**

The PBPK model was constructed in Simcyp using in silico, in vitro, and clinical data and was optimized and verified using clinical data.

**Results:**

The PBPK model described non-linear pharmacokinetics of ipatasertib and captured the magnitude of the observed clinical DDIs. Following repeated doses of 400 mg ipatasertib once daily (QD), the PBPK model predicted a 3.3-fold increase of ipatasertib exposure with itraconazole; a 2–2.5-fold increase with moderate CYP3A4 inhibitors, erythromycin and diltiazem; and no change with a weak CYP3A4 inhibitor, fluvoxamine. Additionally, in the presence of strong or moderate CYP3A4 inducers, rifampicin and efavirenz, ipatasertib exposures were predicted to decrease by 86% and 74%, respectively. As a perpetrator, the model predicted that ipatasertib (400 mg) caused a 1.7-fold increase in midazolam exposure.

**Conclusion:**

This study demonstrates the value of using a fit-for-purpose PBPK model to assess the clinical DDIs for ipatasertib and to provide dosing strategies for the concurrent use of other CYP3A4 perpetrators or victims.

**Supplementary Information:**

The online version contains supplementary material available at 10.1007/s00280-022-04434-2.

## Introduction

Ipatasertib (GDC-0068) is a potent, highly selective small-molecule inhibitor of the three isoforms of the serine–threonine kinase AKT (Akt1, Akt2, and Akt3) or protein kinase B [1–3]. It was developed to treat cancers, such as breast and prostate cancers, with a high prevalence of PI3K/AKT pathway activation, promoting tumor survival, proliferation, growth and changes in cellular metabolic pathways [4, 5]. Ipatasertib in combination with abiraterone has showed prolonged radiographic progression-free survival compared to placebo with abiraterone in patients with metastatic prostate cancer in a phase 2 clinical study [6]. Currently, multiple clinical studies are ongoing to evaluate ipatasertib in combination with hormonal agents, targeted agents or chemotherapy for the treatment of solid tumors.

Ipatasertib has high solubility (> 10 mg/mL across the pH range of 1.1–7.0) and moderate permeability. Food does not affect exposures of ipatasertib [7]. In first-in-human dose escalation study (25–800 mg), ipatasertib showed rapid oral absorption with median time to peak concentration (*T*_max_) ranging from 0.5 to 3 h [7]. The mean terminal half-life of ipatasertib ranged from 31.9 to 53.0 h at doses above 100 mg [7]. During dose escalation stage, ipatasertib exposures increased with increasing doses and were approximately dose-proportional from 200 to 800 mg following a single dose or multiple doses [7]. Exposure at 100 mg was close to dose-proportional after a single dose and more than dose-proportional increase in exposures was observed at the low doses of 25–50 mg [7]. The absolute bioavailability of ipatasertib was estimated to be 34%. Following a single 200 mg oral dose, approximately 24% unchanged drug was eliminated in feces and 8% in urine, indicating that ipatasertib was primarily eliminated by metabolism (ClinicalTrials.gov Identifier: NCT02390492).

In vitro studies indicated that ipatasertib is a substrate and a competitive and time-dependent inhibitor (TDI) of CYP3A4. The potential drug–drug interaction (DDI) risk of ipatasertib with inhibitors and substrates of CYP3A4 was therefore assessed in clinical studies. Itraconazole, a potent CYP3A4 inhibitor, increased ipatasertib (100 mg single oral dose) area under the curve (AUC) and maximum concentration (*C*_max_) by 5.45-fold and 2.26-fold, respectively [8]. In the presence of ipatasertib (600 mg QD), the AUC and *C*_max_ of midazolam, a sensitive CYP3A4 substrate, increased by 2.22-fold and 1.29-fold, respectively [7]. Given the observed effects of CYP3A4 modulations on the pharmacokinetics (PK) of ipatasertib, additional evaluation of DDI potentials for ipatasertib is crucial to inform on concomitant medication use during ipatasertib treatment at clinically intended dose of 400 mg.

As recommended by guidance from the US Food and Drug Administration (FDA) and European Medicines Agency (EMA) for investigating drug interactions [9, 10], the physiologically based pharmacokinetic (PBPK) modeling and simulation approach has been commonly used to predict the PK and enzyme-mediated DDI, providing dosing recommendations in drug applications [11–13]. The aim of this study was to develop a fit-for-purpose PBPK model for ipatasertib to predict additional untested drug interactions for ipatasertib.

## Methods

**Fig. 1:**
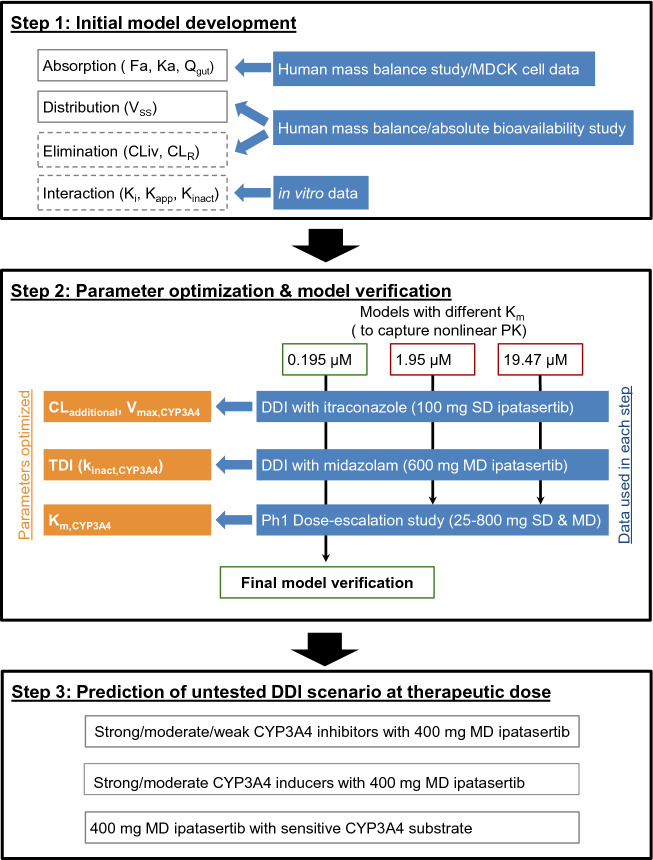
Workflow of ipatasertib PBPK model development, verification and application. *F*_*a*_ fraction absorbed, *K*_*a*_ first-order absorption rate constant, *Q*_*gut*_ a nominal flow in gut model, *V*_*ss*_ volume of distribution at steady state, *CL*_*R*_ renal clearance, *K*_*i*_ concentration of inhibitor that supports half-maximum inhibition, *K*_*app*_ concentration of mechanism-based inhibitor associated with half-maximal inactivation rate, *K*_*inact*_ inactivation rate, *CL*_*additional*_ additional systemic clearance, *MD* multiple dose, *SD* single dose

A PBPK model of ipatasertib was developed using Simcyp population-based absorption, distribution, metabolism, and excretion (ADME) simulator v.18 (Certara, Sheffield, UK) and the model was optimized, verified and applied to predict untested DDIs according to the workflow shown in Fig. 1. The initial model development utilized in silico, in vitro, and clinical PK data. Parameter optimization and verification were performed using clinical PK and DDI data. The verified PBPK model was then used to predict untested DDIs of ipatasertib at the clinically intended dose of 400 mg as the victim or perpetrator of CYP3A4. The ipatasertib PK was comparable between patients with cancer and healthy subjects and the Simcyp healthy volunteer population (“Sim-Healthy Volunteers”) was used in all model development and application steps. For each simulation, the age range, proportion of females, and dosing regimen were set to match the observed ranges in the relevant clinical studies.

### Initial model development

The physiochemical and ADME parameters used for the development of the initial PBPK model are summarized Table S1. A first-order absorption model was used to describe the ipatasertib absorption. The fraction absorbed (fa) was estimated based on the human mass balance study following administration of a single oral dose of 200 mg ipatasertib (ClinicalTrials.gov Identifier: NCT02390492), in which unchanged ipatasertib in feces accounted for 24% of the administered dose. Fa was assumed to be dose-independent since ipatasertib has high solubility (> 10 mg/mL across the pH range of 1.1–7.0) and moderate permeability. The *K*_a_ and a nominal flow in gut model (*Q*_gut_) were predicted within Simcyp using ipatasertib MDCK permeability data. For ipatasertib distribution, a full PBPK model was used. The volume of distribution at steady state (*V*_ss_) was obtained from the clinical absolute bioavailability study (ClinicalTrials.gov Identifier: NCT02390492). A tissue to plasma partition coefficient (*K*_p_) scalar was applied to match the predicted *V*_ss_ (predicted with in Simcyp using Method 3, Rodgers et al. + ion membrane permeability) to the observed value. The IV clearance determined in the clinical absolute bioavailability study was used (ClinicalTrials.gov Identifier: NCT02390492). CYP3A4 was assigned to account for 100% of hepatic clearance (CL_H_), as in vitro study suggested that ipatasertib was primarily metabolized by CYP3A4 [8]. The renal clearance (CL_R_) was calculated using Eq. 1:

1 $${\text{CL}}_{{\text{R}}} = {\text{Ae}}/{\text{AUC}}_{0 - \infty } ,$$

in which Ae is the amount of unchanged drug excreted in urine following a single oral dose of 200 mg ipatasertib in healthy subjects and AUC_0–∞_ is the area under the concentration–time curve extrapolated to infinity from the same clinical study (ClinicalTrials.gov Identifier: NCT02390492). The CYP3A4 inhibition parameters for competitive inhibition and TDI determined using in vitro assays (Data on file) were incorporated into the model.

To assess the initial model, the PK of ipatasertib following a single intravenous (IV) dose of 0.08 mg or oral dose of 200 mg was simulated separately with 10 trials containing 8 subjects per trial over 3 days and compared with the clinical PK data in the absolute bioavailability study. The age range of 21–41 and the proportion of females of 0 were used in the simulation.

The performance of the PBPK model that incorporated TDI of CYP3A4 was also evaluated for its ability to explain the nonlinearity in exposures at the lower doses. In this case, ipatasertib PK was simulated following single and multiple oral doses over a range of 25–800 mg and was compared to the observed clinical data in the dose escalation study [7]. The ipatasertib PK (25–800 mg ipatasertib QD) was simulated with 10 trials containing 29 subjects per trial over 8 days. The age range of 32–73 and the proportion of females of 0.55 were used in the simulation.

### Optimization and verification of model parameters

In the clinical dose escalation study (25–800 mg), more than a dose-proportional increase of ipatasertib exposure was observed at lower doses (25–50 mg) after single and multiple doses. The initial model with consideration of TDI using the observed IV clearance failed to describe the observed nonlinearity of ipatasertib PK. Therefore, parameters related to ipatasertib metabolism were optimized to capture nonlinear PK as well as clinical DDI data in a stepwise manner (Fig. 1). Briefly, *K*_m_ and *V*_max_ of CYP3A4 were first optimized by capturing the clinical DDI between ipatasertib and itraconazole without incorporating CYP3A4 TDI parameters in the model. Three models with different *K*_m_ values were developed in parallel to ensure the observed nonlinear PK of ipatasertib could be properly described in the later step. Then, to ensure the model could properly describe the inhibitory impact of ipatasertib on CYP3A4, the inactivation rate (*K*_inact_) of CYP3A4 was optimized in each model by capturing the clinical DDI between midazolam and ipatasertib. Lastly, a model incorporating the optimized *K*_m_, *V*_max_ and *K*_inact_, and capturing the nonlinear PK of ipatasertib at lower doses was selected from three developed models as the final model. Details are described in the subsequent sections.

#### Simulation of DDI between ipatasertib and itraconazole

To determine the fraction metabolized by CYP3A4, *K*_m_ and *V*_max_ of CYP3A4 and additional hepatic clearance (CL_additional_) were optimized by capturing the observed DDI between ipatasertib and itraconazole.

Briefly, starting from the in vitro measured *K*_m,CYP3A4_ of 19.47 μM, a sensitivity analysis was conducted with 10- and 100-fold lower values of *K*_m_,_CYP3A4_ (1.95 μM and 0.195 μM) used in the simulations. The Simcyp retrograde calculator in the recombinant CYP module was then used to calculate the intrinsic clearance of CYP3A4 (CL_int,CYP3A4_) (assuming CYP3A4 contributes to 100% Hep met CL) from the observed IV clearance and CL_R_ and assigned CL_addtional_, and from there, *V*_max,CYP3A4_ was calculated using Eq. 2:

2 $$V_{{\text{max,CYP3A4}}} = CL_{{\text{int,CYP3A4}}} \times K_{{\text{m,CYP3A4}}} .$$

A total of three models (three sets of *K*_m,CYP3A4_, *V*_max,CYP3A4_, and CL_additional_) that can capture the observed DDI between ipatasertib and itraconazole, were used to optimize inhibitory parameters in the subsequent step. Since ipatasertib was given as a low single dose (100 mg) in this DDI study, the impact of auto-inhibition caused by TDI was expected to be minimal, and parameters related to TDI were not included in this step. The Simcyp default compound file of itraconazole (fasted, solution) and OH-itraconazole was used for the simulation. The interaction between ipatasertib (100 mg, single dose, starting on Day 5) and itraconazole (200 mg QD of 9 doses, starting on Day 1) was simulated with 10 trials containing 15 subjects per trial over 12 days. The age range of 22–52 and the proportion of females of 0.67 were used in the simulation.

#### Simulation of DDI between ipatasertib and midazolam

DDI between ipatasertib and midazolam was simulated using the Simcyp default compound file of midazolam and compared with observed data from a clinical study conducted in patients with cancer [7]. The in vitro measured CYP3A4 inhibitory parameters of ipatasertib, *K*_i_ of 4.4 μM, *K*_app_ of 9.66 μM and *K*_inact_ of 2.6 1/h, were used in the ipatasertib model. Briefly, the interaction between midazolam (2 mg, single oral dose, starting on Day 8) and ipatasertib (600 mg QD of 8 doses, starting on Day 1) was simulated with 10 trials containing 13 subjects per trial over 8 days. The age range of 37–76 and the proportion of females of 0.69 were used in the simulation.

Initially, the DDI between midazolam and ipatasertib was consistently over-predicted. Therefore, sensitivity analyses of inhibitory parameters, where only one parameter (*K*_i_ or *K*_inact_) varied at a time, were performed for all three models described above. Based on results of sensitivity analyses, *K*_inact_ was optimized in each model to capture the clinical DDI between midazolam and ipatasertib.

#### Simulation of ipatasertib PK following single and multiple oral doses

Three models with optimized enzyme kinetic (*V*_max_ and *K*_m_) and inhibitory (*K*_inact_) parameters of CYP3A4 and CL_additional_ were established as described above with the rest of the input parameters remaining the same, and each model predicted the clinical DDI between ipatasertib and itraconazole or midazolam. Lastly, to estimate the value of *K*_m_,_CYP3A4_ while capturing the nonlinearity of ipatasertib PK, the ipatasertib PK following single and multiple doses at the dose range of 25–800 mg was simulated using each model and compared to the PK profile of observed clinical studies. The parameters used in the final model that described the nonlinearity of ipatasertib PK are listed in Table 1.

**Table 1:** Input parameters for the final ipatasertib PBPK model

Parameter	Value	Reference
MW (g/mol)	458	In-house data
LogP	3	In-house data
Compound type	Diprotic base	In-house data
*p*Ka1	9	In-house data
*p*Ka2	4.9	In-house data
*B*/*P* ratio	1.43	Mean for ipatasertib at concentration from 0.1 to 40 µM; Data on file
*f*_u,plasma_	0.63	Mean for ipatasertib at concentration from 0.1 to 40 µM; Data on file
*Absorption-1st-order absorption model*		
*F*_a_	0.76	Data on file
*K*_a_ (1/h)	0.76	Predicted
*f*_u,gut_	1	Simcyp default value
*Q*_gut_ (L/h)	9.28	Predicted
MDCK (10^–6^ cm/s)	3.55	In-house data
Permeability predication scalar	3.89	Predicted with multiple references^a^
*P*_eff, man_ (10^−4^ cm/s)	1.74	Predicted
*Distribution-full PBPK model*		
*V*_ss_ (L/kg)	39.13	Data on file
*K*_p_ scalar	4.47	Assigned
*Elimination-enzyme kinetics-recombinant*		
*V*_max,CYP3A4_ (pmol/min/pmol)	0.135	Optimized
*K*_m,CYP3A4_ (µM)	0.195	Optimized
*f*_u,mic_	1	Simcyp default value
CL_additional_ (L/h)	2.7	Optimized
CL_R_ (L/h)	19.3	Data on file
*Interaction-CYP3A4 inhibition*		
Competitive inhibition		
*K*_i_ (µM)	4.4	Data on file
*f*_u,mic_	1	Simcyp default value
Time-dependent inhibition		
*K*_app_ (µM)	9.66	Data on file
*K*_inact_ (1/h)	0.17	Data on file
*f*_u,mic_	1	Simcyp default value

*MW* molecular weight, *B/P* blood-to-plasma partition ratio, *f*_*u,plasma*_ fraction unbound in plasma, *F*_*a*_ fraction absorbed, *K*_*a*_ first-order absorption rate constant, *f*_*u,gut*_ unbound fraction of drug in enterocytes, *Q*_*gut*_ a nominal flow in gut model, *MDCK* Madin-Darby Canine Kidney, *P*_*eff,man*_ human jejunum effective permeability, *V*_*ss*_ volume of distribution at steady state, *K*_*p*_ tissue to plasma partition coefficient, *CL*_*additional*_ additional systemic clearance, *CL*_*R*_ renal clearance, *K*_*i*_ concentration of inhibitor that supports half-maximum inhibition, *f*_*u,mic*_ fraction of unbound drug in the in vitro microsomal incubation, *K*_*app*_ concentration of mechanism-based inhibitor associated with half-maximal inactivation rate, *K*_*inact*_ inactivation rate

^a^Reference (*P*_app_ 10^–06^ cm/s): cimetidine—1; atenolol—0.1; propranolol—20.9; verapamil—11.2; midazolam—18.8; metoprolol—24.8

### Verification of ipatasertib PBPK model

To verify the performance of the final PBPK model, ipatasertib PK was simulated and compared with observations from two clinical studies that were not used for model development and optimization. In the first clinical study, AUC and *C*_max_ of ipatasertib decreased by approximately 50% and 53% in the presence of enzalutamide (a strong CYP3A4 inducer) in patients with prostate cancer [14, 15]. The interaction between ipatasertib (400 mg QD of 43 doses, starting on Day 1) and enzalutamide (160 mg QD of 35 doses, starting on Day 9) was simulated with 10 trials containing 23 subjects per trial over 44 days. The age range of 56–83 with the proportion of females of 0 was used in the simulation. A published enzalutamide PBPK model [16] was used and the input parameters of the enzalutamide PBPK model are listed in Table S2.

The second study used for model verification is the study of ipatasertib co-administered with palbociclib (a substrate and TDI of CYP3A4) [17], in which ipatasertib AUC and *C*_max_ increased by approximately 63% and 44% in the presence of palbociclib in patients with breast cancer (ClinicalTrials.gov Identifier: NCT04060862). Briefly, the interaction between ipatasertib (300 mg QD of 22 doses, starting on Day 1) and palbociclib (125 mg QD of 15 doses, starting on Day 8) was simulated with 10 trials containing 9 subjects per trial over 23 days. A published palbociclib PBPK model [18] and the age range of 49–67 with the proportion of females of 1 were used in the simulation. The input parameters of the palbociclib PBPK model are summarized in Table S3.

### Application of the ipatasertib PBPK model

The verified ipatasertib PBPK model was used to assess CYP3A4-mediated DDIs between ipatasertib at the intended therapeutic dose of 400 mg with strong, moderate or weak CYP3A4 inhibitors or inducers and a sensitive CYP3A4 substrate. In addition, DDIs between 200 mg ipatasertib and moderate CYP3A4 inhibitors were simulated and compared with 400 mg ipatasertib alone to assess the adequacy of dose reduction when concurrent use of moderate CYP3A4 inhibitors cannot be avoided. An age range of 20–95, the age range for the default cancer population, and a proportion of females of 0.5 were used in the Simcyp default healthy volunteer population for the simulation.

#### Effect of CYP3A4 inhibitors on ipatasertib PK

To evaluate the effects of strong (itraconazole), moderate (erythromycin and diltiazem) and weak (fluvoxamine) CYP3A4 inhibitors on ipatasertib PK, Simcyp default compound files of itraconazole, erythromycin, diltiazem, and fluvoxamine were used in the simulation with the ipatasertib PBPK model. Briefly, the interaction between ipatasertib (400 mg QD of 21 doses starting on Day 1) and CYP3A4 inhibitor [itraconazole 200 mg QD of 21 doses; erythromycin 500 mg three times daily (TID) of 63 doses; diltiazem 120 mg twice daily (BID) of 42 doses; fluvoxamine 100 mg QD of 21 doses, starting on Day 1] was simulated with 10 trials containing 10 subjects per trial over 21 days.

To evaluate the impact of reducing the ipatasertib dose from 400 to 200 mg when co-administered with moderate inhibitors, simulations were conducted to evaluate interactions between ipatasertib (200 mg) and erythromycin or diltiazem with the remaining trial parameters maintained as described above.

#### Effect of CYP3A4 inducers on ipatasertib PK

To access the effect of CYP3A4 inducers on ipatasertib exposures, the PK of ipatasertib was simulated in the presence of rifampicin and efavirenz, strong and moderate inducers of CYP3A4, respectively. Simcyp default compound files of rifampicin and efavirenz were used in the simulation. Briefly, the interaction between ipatasertib (400 mg QD of 21 doses starting on Day 1) and CYP3A4 inducer (rifampin 600 mg QD of 21 doses; efavirenz 600 mg QD of 21 doses, starting on Day 1) was simulated with 10 trials containing 10 subjects per trial for 21 days.

#### Effect of ipatasertib on PK of a sensitive CYP3A4 substrate

To evaluate the effect of ipatasertib (at the clinically intended dose of 400 mg) on the PK of a sensitive CYP3A4 substrate, the DDI between midazolam and ipatasertib was simulated. The Simcyp default compound file of midazolam was used in the simulation. Briefly, the interaction between midazolam (2 mg, single dose, starting on Day 8) and ipatasertib (400 mg QD of 8 doses starting on Day 1) was simulated with 10 trials containing 10 subjects per trial over 8 days.

## Results

### Initial ipatasertib PBPK model development

The initial PBPK model was able to describe the observed ipatasertib PK following a single IV dose of 0.08 mg (Figure S1a). However, the PBPK model did not adequately capture the observed PK after a single oral dose of 200 mg ipatasertib in the absolute bioavailability study or the observed nonlinear PK following single and multiple oral doses at the dose range of 25–800 mg in the dose escalation study (Figures S1b and c). At steady state, the observed oral clearance of ipatasertib (Dose/AUC_0–∞_) decreased at the dose range of 25–50 mg, approached linearity at 100 mg and became linear at 200–800 mg after single and multiple doses. In contrast, the model predicted no change in oral clearance across all dose levels after the first dose and at steady state (Figures S1b and c), even though the model incorporated TDI using in vitro parameters.

### Optimization and verification of model parameters

The initial model incorporating CYP3A4 inhibitory parameters obtained from in vitro studies did not predict the nonlinear PK of ipatasertib well. Since ipatasertib is primarily metabolized by CYP3A4, the observed nonlinearity could likely be mainly caused by the saturation of CYP3A4 in the gut and liver. To capture the saturation of CYP3A4 and the DDI between ipatasertib and itraconazole, enzyme kinetic parameters (*K*_m_ and *V*_max_) of CYP3A4 were used instead of CL_iv_. The stepwise optimization strategy is illustrated in Fig. 1.

#### Simulation of DDI between ipatasertib and itraconazole

To capture the observed DDI between ipatasertib and itraconazole, CL_additional_ and *V*_max_ were optimized with fixed *K*_m_ values of 0.195, 1.95, or 19.47 μM. Three models were developed in parallel (TDI parameters were not included) and simulated with the optimized CL_additional_ (L/h) of 2.7, 11.8 and 13.7 and *V*_max_ (pmol/min/pmol) of 0.135, 1 and 9.37 corresponding to *K*_m_ of 0.195, 1.95, and 19.47 μM, respectively. All three models predicted ipatasertib PK and the DDI between ipatasertib and itraconazole (data not shown).

#### Simulation of DDI between ipatasertib and midazolam

The model using in vitro measured competitive inhibition and TDI parameters of CYP3A4 over-predicted the DDI between ipatasertib and midazolam. As shown in Figure S2a, changes in competitive inhibition (*K*_i_) had a minor effect while changes in TDI (*K*_inact_) had a substantial impact on the predicted magnitude of DDI (Figure S2b). Therefore, TDI parameters were included and *K*_inact_ was optimized to capture the observed DDI between midazolam and ipatasertib. The optimized values for *K*_inact_ (1/h) were 0.17, 0.33 and 0.41 corresponding to *K*_m_ (μM) values of 0.195, 1.95 and 19.47, respectively. All three models predicted the magnitude of the change in midazolam exposure in the presence of ipatasertib (data not shown for models with *K*_m_ of 19.47 μM, and 1.95 μM; the model with *K*_m_ of 0.195 µM is shown in Table 2).

**Table 2:** Summary of model-simulated and clinically observed pharmacokinetic parameters in studies with ipatasertib as the victim or perpetrator of CYP3A4

Clinical scenario	*C*_max_ (ng/ml)			AUC (ng* h/ml)			*T*_max_ (h)		
	Observation	Prediction	*P*/*O*	Observation	Prediction	*P*/*O*	Observation	Prediction	*P*/*O*
Midazolam (2 mg single oral dose) administered with and without ipatasertib ( 600 mg QD)									
Midazolam	15.0 (51.6%)	7.6 (69%)	0.51	39.3 (58.4%)	23.0 (70%)	0.59	0.50 (0.50–1.00)	0.65 (0.31–1.18)	1.30
Midazola + ipatasertib	19.3 (39.1%)	13.1(79%)	0.68	87.1 (53.3%)	51.0 (86%)	0.59	1.48 (1.00–2.00)	0.71 (0.31–1.30)	0.48
Midazolam ratio	1.29 (0.97–1.71)	1.72 (1.67–1.77)	1.33	2.22 (1.57–3.12)	2.22 (2.10–2.33)	1.00			
Ipatasertib (100 mg single oral dose) administered with and without itraconazole (200 mg QD)									
Ipatasertib	44.9 (35.9%)	40.0 (39%)	0.89	327 (26.4%)	329 (49%)	1.01	1.07 (0.50–3.03)	1.46 (0.77–2.32)	1.36
Ipatasertib + itraconazole	102 (34.1%)	68 (30%)	0.67	1780 (22.6%)	1793 (38%)	1.01	2.05 (1.00–6.00)	2.27 (1.51–3.75)	1.11
Ipatasertib ratio	2.26 (1.83–2.80)	1.69 (1.65–1.73)	0.75	5.45 (4.96–5.98)	5.45 (5.30–5.62)	1.00			
Ipatasertib (400 mg QD) administered with and without enzalutamide (160 mg QD)									
Ipatasertib	284 (66.5%)	289 (45%)	1.01	2170 (53.8%)	3172 (62%)	1.46	1.85 (0.63–4.00)	2.14 (1.23–3.36)	1.16
Ipatasertib + enzalutamide	133 (60.0%)	169 (70%)	1.27	1083 (34.6%)	1204 (122%)	1.11	1.00 (0.25–24.07)	1.60 (0.65–3.20)	1.60
Ipatasertib ratio	0.47 (0.35–0.63)	0.58 (0.54–0.64)	1.23	0.50 (0.40–0.62)	0.38 (0.34–0.43)	0.76			
Ipatasertib (300 mg QD) administered with and without palbociclib (125 mg QD)									
Ipatasertib	314 (51.4%)	239 (44%)	0.76	2513 (49.4%)	2363 (61%)	0.94	1.50 (0.50–4.00)	1.68 (0.85–3.46)	1.12
Ipatasertib + palbociclib	437 (41.1%)	295 (46%)	1.80	4000 (34.8%)	3496 (63%)	0.87	2.00 (0.50–4.00)	1.85 (0.86–3.51)	0.93
Ipatasertib ratio	1.44 (1.10–1.88)	1.23 (1.10–1.38)	0.85	1.63 (1.33–1.99)	1.48 (1.25–1.75)	0.91			

AUC and *C*_max_ are reported as geometric mean (CV%); *T*_max_ is reported as median (range: minimum–maximum); AUC and *C*_max_ ratio are reported as geometric mean ratio (90% confidence interval); *P/O* predicted/observed values; In the first two studies, listed AUC is AUC_0–∞_, while in the last two studies AUC is AUC_0–24 h_; Shown here is the simulation using the final model

#### Simulation of ipatasertib PK following single and multiple oral doses

**Fig. 2:**
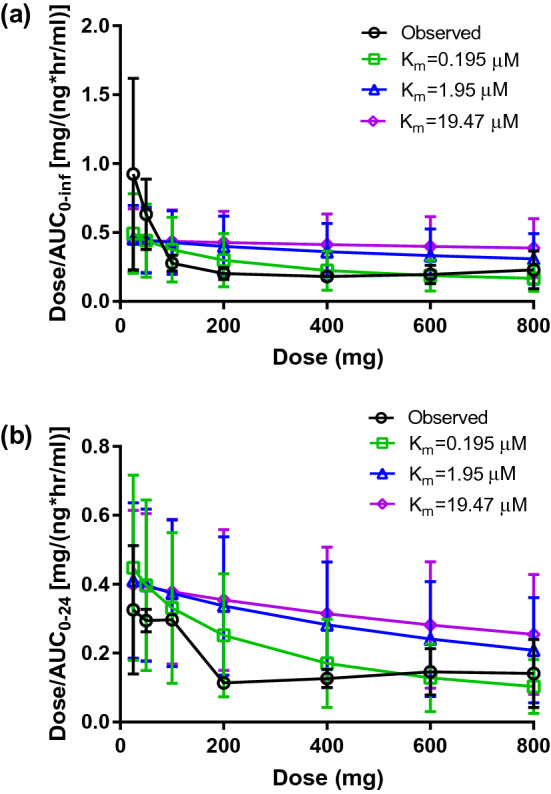
Observed and predicted changes of ipatasertib oral clearance (mean ± SD) following **a** a single dose and **b** at steady-state

**Fig. 3:**
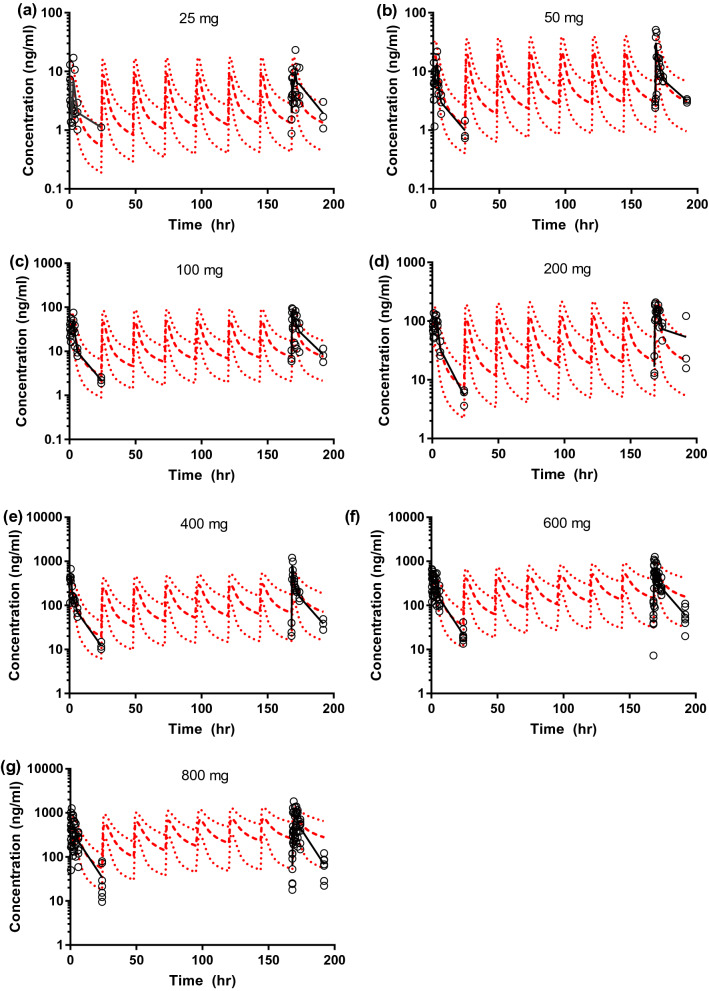
Observed and predicted plasma concentration–time profiles of ipatasertib following single and multiple oral doses. Solid black line—observed mean plasma concentrations; circles—observed individual plasma concentrations; dashed red lines—predicted mean, 95th and 5th percentile of plasma concentrations

The PK profiles of ipatasertib following single and multiple doses (25–800 mg) were simulated using the three optimized models. Among the models that described the DDIs mentioned above, the model with *K*_m_ of 0.195 µM best described the observed trend of nonlinear PK of ipatasertib at the dose range of 25–800 mg (Fig. 2). The simulated PK of ipatasertib using the model with *K*_m_ of 0.195 µM matched the observed PK reasonably well, especially around the clinically intended dose of 400 mg (Fig. 3 and Table S4). Therefore, the model with *K*_m_ of 0.195 µM was considered to be the final model.

**Fig. 4:**
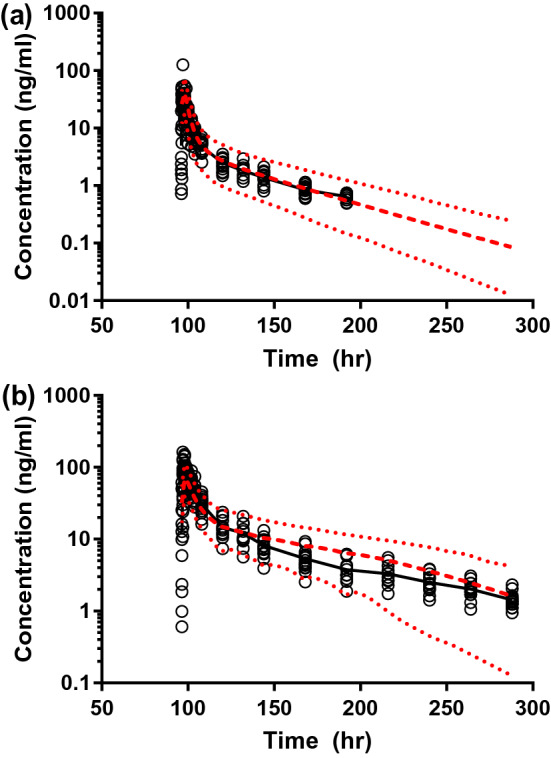
Simulated and observed plasma concentration–time profiles of ipatasertib following a 100 mg single oral dose in the **a** absence and **b** presence of 200 mg itraconazole. Solid line—observed mean plasma concentrations; circles—observed individual plasma concentrations; dashed line—predicted mean plasma concentrations, 95th and 5th percentile. Shown here is the simulation of the finalized model

Using the final model, the DDI between itraconazole and ipatasertib was simulated in an iterative learn-and-confirm approach. The simulated magnitude of DDI was consistent with the observed magnitude, and the simulated ipatasertib PK profile was in good agreement with the observed PK profile of 100 mg ipatasertib in the absence and presence of itraconazole (Fig. 4 and Table 2). Additionally, the simulated itraconazole PK profile was in agreement with the clinical observation (Figure S3a). The final model also predicted the observed PK of ipatasertib following a single IV or oral dose in the study that was used for initial model development (Figure S3b and c).

### Verification of ipatasertib PBPK model

The verification of the ipatasertib PBPK model was performed using clinical data from two independent studies. In the first study, the model predicted 42% and 62% decrease in *C*_max_ and AUC of ipatasertib in the presence of enzalutamide (a strong CYP3A4 inducer), which was comparable to the observed 53% and 50% decrease in *C*_max_ and AUC (Table 2). In the second study, in the presence of palbociclib (a substrate and TDI of CYP3A4), *C*_max_ and AUC of ipatasertib were predicted to increase by 23% and 48%, respectively (Table 2). The predicted changes in ipatasertib exposures were slightly lower but still comparable to the observed 44% and 63% increase in ipatasertib exposures in the presence of palbociclib (Table 2). Moreover, the simulated exposures of enzalutamide and palbociclib were in a good agreement with the clinical observations as described in Table S5.

### Application of the ipatasertib PBPK model

#### Effect of CYP3A4 inhibitors on ipatasertib PK

**Fig. 5:**
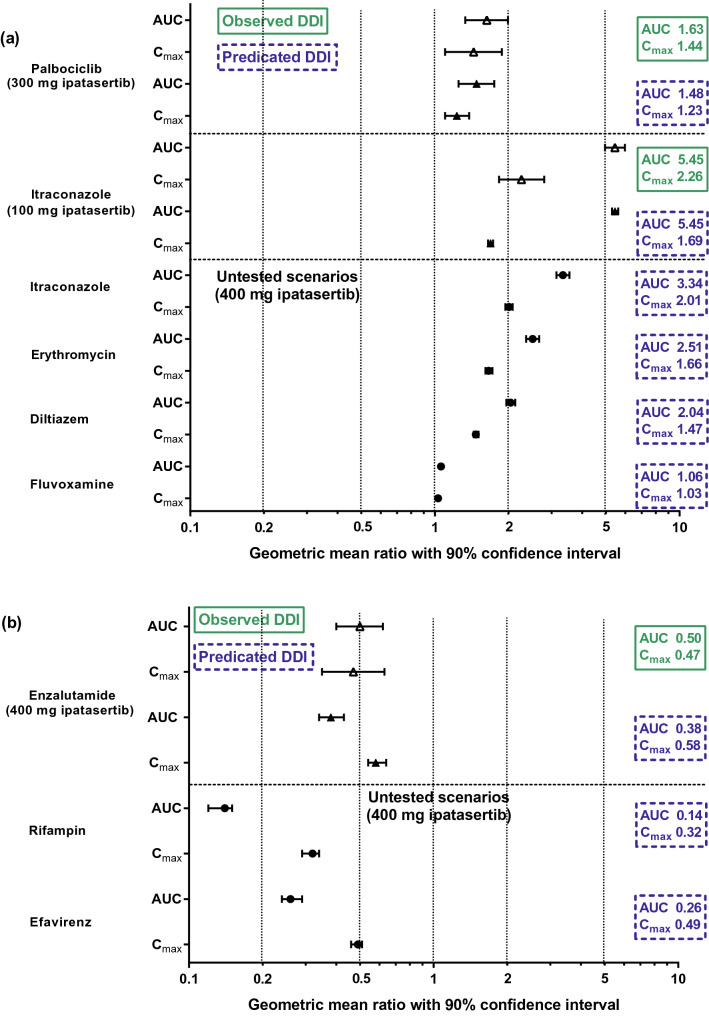
Observed and simulated ipatasertib AUC and *C*_max_ ratios with various CYP3A4 inhibitors and inducers

The impact of strong (itraconazole), moderate (erythromycin and diltiazem), and weak (fluvoxamine) inhibitors of CYP3A4 on the PK of ipatasertib (400 mg) was assessed using the developed PBPK model. The simulation showed that the fold-change in ipataserib AUC was 3.34, 2.51, 2.04, and 1.06 for itraconazole, erythromycin, diltiazem, and fluvoxamine, respectively. The fold-change in ipataserib *C*_max_ was predicted to be 2.01, 1.66, 1.47, and 1.03 for itraconazole, erythromycin, diltiazem, fluvoxamine, respectively (Table 3). The observed and simulated ipatasertib AUC, *C*_max_ ratios and 90% confidence intervals (CI) with various CYP3A4 inhibitors are shown in Fig. 5a.

**Table 3:** Simulation of DDIs between 400 mg ipatasertib QD and CYP3A4 inhibitors, inducers or a sensitive CYP3A4 substrate

CYP3A4 inhibitor	Inhibition of CYP3A4	Fold change of ipatasertib AUC (90% CI)	Fold change of ipatasertib *C*_max_ (90% CI)
Itraconazole solution 200 mg QD	Strong	3.34 (3.14–3.55)	2.01 (1.94–2.08)
Erythromycin 500 mg TID	Moderate	2.51 (2.36–2.67)	1.66 (1.61–1.72)
Diltiazem 120 mg BID	Moderate	2.04 (1.96–2.13)	1.47 (1.44–1.51)
Fluvoxamine 100 mg QD	Weak	1.06 (1.05–1.06)	1.03 (1.03–1.03)

CYP3A4 inducer	Induction of CYP3A4	Fold change of ipatasertib AUC (90% CI)	Fold change of ipatasertib *C*_max_ (90% CI)
Rifampin 600 mg QD	Strong	0.14 (0.12–0.15)	0.32 (0.29–0.34)
Efavirenz 600 mg QD	Moderate	0.26 (0.24–0.29)	0.49 (0.46–0.51)

Sensitive CYP3A4 substrate		Fold change of midazolam AUC (90% CI)	Fold change of midazolam *C*_max_ (90% CI)
Midazolam 2 mg single dose		1.69 (1.63–1.75)	1.49 (1.45–1.52)

*Fold change of ipatasertib AUC or C*_*max*_ geometric mean ratio of AUC of ipatasertib in the presence of inhibitor/inducer to AUC or *C*_max_ in the absence of inhibitor/inducer; *Fold change of midazolam AUC or C*_*max*_ geometric mean ratio of AUC of midazolam in the presence of ipatasertib to AUC or *C*_max_ in the absence of ipatasertib

Additionally, simulations showed that the steady-state AUC of ipatasertib at a reduced dose of 200 mg administered concurrently with moderate CYP3A4 inhibitors erythromycin or diltiazem was comparable to the predicted steady-state AUC of ipatasertib at the clinically intended dose of 400 mg without inhibitors (Table S6).

#### Effect of CYP3A4 inducers on ipatasertib PK

The impact of strong (rifampin) and moderate (efavirenz) inducers of CYP3A4 on the PK of ipatasertib (400 mg) was assessed using the PBPK model. The simulation showed the fold-change in ipatasertib AUC was 0.14 and 0.26 for rifampin and efavirenz, respectively, while the fold-change in ipatasertib *C*_max_ was 0.32 and 0.49 for rifampin and efavirenz, respectively (Table 3 and Fig. 5b).

#### Effect of ipatasertib on PK of a sensitive CYP3A4 substrate

The potential impact of 400 mg ipatasertib on the PK of midazolam, a sensitive CYP3A4 substrate, was evaluated using the ipatasertib PBPK model. The model predicted a fold-change in midazolam AUC of 1.69 (90% CI 1.63–1.75) and a fold-change in *C*_max_ of 1.49 (90% CI 1.45–1.52) when co-administered with 400 mg ipatasertib (Table 3).

## Discussion

A PBPK model of ipatasertib was developed using both in vitro and clinical data to assist with DDI predictions. The key clinical studies that helped in a top–down approach for model development and optimization of in vitro parameters included ADME studies (absolute bioavailability and mass balance studies), a dose escalation study showing nonlinear PK, and DDI studies with midazolam and itraconazole. The clinical data were used in a learn-and-confirm iterative process to optimize in vitro parameters of CYP3A4-mediated metabolism (*K*_m_ and *V*_max_) and TDI (*K*_inact_). The stepwise parameter optimization process is described in Fig. 1. After initial model development, models with three different *K*_m,CYP3A4_ values were optimized in parallel for CL_additional_ and *V*_max,CYP3A4_ using clinical PK data from the itraconazole DDI study. The reason for using this clinical DDI data as the initial step of optimization was that the effect of TDI was expected to be minimal after a single dose of 100 mg ipatasertib, thereby making a separate optimization of the CYP3A4 enzyme kinetic parameters feasible. Further, *K*_inact,CYP3A4_ was optimized with the goal of replicating results from the DDI study with midazolam. This optimization approach is not uncommon as the magnitude of DDI is often overestimated using in vitro TDI parameters obtained from human liver microsomes [19].

There were several discrepancies between the optimized parameter values and in vitro values. The model with *K*_m,CYP3A4_ that was lower than the measured in vitro value was selected as the final model to capture the nonlinear PK and the clinical DDI. This optimized *K*_m_ could be considered as a value combining the effect of fraction of unbound drug in the in vitro microsomal incubation and an extrapolation factor that accounting for in vitro to in vivo system translation. Similar explanations could be also applied to the optimized *K*_inact,CYP3A4_. In fact, the extrapolation factor has been used to explain the difference between the in vitro and in vivo system to match the clinical observations for CYP-mediated clearance in multiple studies [20–22]. Moreover, based on the current clinical observations and the simulation results, the nonlinear PK observed at low dose levels was likely due to the CYP3A4 saturation rather than TDI of CYP3A4. However, it is possible that other mechanistic factors could also contribute to the nonlinear PK and the optimized enzyme kinetic and inhibitory parameters could be the net values accounting for those factors. In vitro studies showed that ipatasertib was also a substrate of P-glycoprotein (P-gp) and the saturation of P-gp in liver and intestine may contribute to the observed nonlinearity. However, the available clinical data cannot distinguish the contribution of P-gp from CYP3A4 to the observed nonlinearity. Only 2.9–5.8% of unchanged ipataseritb was found in the bile of rats and monkeys dosed with 10 mg/kg and 5 mg/kg ipatasertib, indicating the biliary elimination may be negligible and the likelihood of the P-gp saturation in liver is low. In addition to these points, although ipatasertib is a weak CYP3A4 inducer in vitro, the induction was not incorporated into the model. This is due to the difficulty in differentiating between the inhibition and induction from the clinical data and that the PBPK model parameters were optimized to represent a net inhibition effect.

This PBPK model was verified with two independent clinical studies, in which the ipatasertib PK profiles changed in the presence of enzalutamide, a strong CYP3A4 inducer or palbociclib, a substrate and TDI of CYP3A4. The simulation of the DDIs was comparable to the clinical observations, indicating the capability of the ipatasertib PBPK model in predicting the interaction with CYP3A4 inducers and inhibitors. Notably, in the presence of enzalutamide, ipatasertib exposures decreased approximately 50%, while the predicted decreases of exposures in the presence of rifampin and efavirenz were 86% and 74%, respectively. This could be explained by the fact that enzalutamide may be also a substrate and an inhibitor of CYP3A4 as suggested by the in vitro and clinical data [14, 23]. Moreover, in the presence of ipatasertib, a competitive and TDI of CYP3A4, the simulated palbociclib exposures matched the observations well, indicating the ipatasertib PBPK model reasonably described the inhibitory effect of ipatasertib. This fit-for-purpose PBPK model of ipatasertib was considered adequate for DDI prediction as several clinical studies were used in calibrating and verifying the model and key DDIs were captured.

As a substrate of CYP3A4, a 5.45-fold increase in AUC was observed with 100 mg ipatasertib in the presence of itraconazole. However, the DDI magnitude was expected to be lower at the clinically intended dose of 400 mg as the competition for CYP3A4 is typically concentration-dependent, which was confirmed using PBPK modeling with a predicted 3.34-fold increase in AUC. The exposure of 100 mg ipatasertib in the presence of the strong CYP3A4 inhibitor itraconazole was comparable to the exposure of ipatasertib alone at the clinically intended dose of 400 mg. Additionally, the AUC was predicted to increase by 2–2.5-fold in the presence of moderate inhibitors. The simulation showed that 200 mg ipatasertib in the presence of moderate CYP3A4 inhibitors had comparable exposures as 400 mg ipatasertib taken alone, suggesting if the concurrent use of moderate CYP3A4 inhibitors is unavoidable, ipatasertib could be reduced to 200 mg. Based on the simulation, weak inhibitors did not appear to affect exposures of ipatasertib appreciably, suggesting dose adjustment is not needed when ipatasertib is administered with weak CYP3A4 inhibitors. The simulation also showed that strong and moderate inducers of CYP3A4 could decrease ipatasertib AUC by 86% and 74% and *C*_max_ by 68% and 51%, suggesting co-medications that are strong and moderate CYP3A4 inducers should be avoided because they may reduce the efficacious exposures of ipatasertib. Together, these simulations provided a model-based dosing strategy to maintain safe and efficacious plasma concentrations of ipatasertib when co-administration with CYP3A4 inhibitors or inducers is needed. In the clinical DDI study with midazolam, ipatasertib administered at 600 mg caused a 2.22-fold increase in midazolam AUC. Using the PBPK model, simulations of midazolam DDI with 400 mg ipatasertib confirmed that ipatasertib is a weak CYP3A4 inhibitor at 400 mg dose with a < 2-fold increase of AUC predicted for midazolam.

In summary, in vitro and clinical PK and DDI data were used to develop, optimize and verify a fit-for-purpose ipatasertib PBPK model. Based on the model prediction, at the clinically intended dose of 400 mg, ipatasertib is a weak CYP3A4 inhibitor, dose adjustment may be needed when chronic co-administration with moderate and strong inhibitors of CYP3A4 is needed and the concurrent use of strong CYP3A4 inducers may need to be avoided. Together with observed clinical studies, this work will provide support for safe and effective use of ipatasertib when given in combination with various modulators and substrates of CYP3A4. The PBPK model helps reduce the number of clinical DDI studies needed to characterize CYP3A4-mediated DDI with ipatasertib and provides an ethical benefit for patients with cancer.

## Supplementary Information

Below is the link to the electronic supplementary material.

Supplementary file1 (DOCX 34 KB)

Supplementary file2 (PDF 270 KB)

Supplementary file3 (PDF 202 KB)

Supplementary file4 (PDF 563 KB)

## Data Availability

The datasets generated during and/or analyzed during the current study are available from the corresponding authors on reasonable request.
