# Supplementary material for: Assessment of cytochrome P450 3A4-mediated drug–drug interactions for ipatasertib using a fit-for-purpose physiologically based pharmacokinetic model
Source: Cancer Chemother Pharmacol. 2022 Apr 15;89(5):707–20. doi: 10.1007/s00280-022-04434-2 (PMC9054915; doi:10.1007/s00280-022-04434-2)
Supplement: Supplementary file 1 — Supplementary file1 (DOCX 34 KB) [file 280_2022_4434_MOESM1_ESM.docx]

**Table S1. Input parameters for the initial ipatasertib PBPK model**

| **Parameter** | **Value** | **Reference** |
| --- | --- | --- |
| MW (g/mol) | 458 | In house data |
| LogP | 3 | In house data |
| Compound type | Diprotic base | In house data |
| pKa1 | 9 | In house data |
| pKa2 | 4.9 | In house data |
| B/P ratio | 1.43 | Mean for ipatasertib at concentration from 0.1-40 µM; Data on file |
| f_u, plasma_ | 0.63 | Mean for ipatasertib at concentration from 0.1-40 µM; Data on file |
| ***Absorption-1st order absorption model*** | | |
| F_a_ | 0.76 | [Data](#z337ya) on file |
| K_a_ (1/h) | 0.76 | Predicted |
| f_u, gut_ | 1 | Simcyp default value |
| Q_gut_ (L/h) | 9.28 | Predicted |
| MDCK (10^-6^ cm/s) | 3.55 | In house data |
| Permeability predication scalar | 3.89 | Predicted with multiple references^a^ |
| P_eff, man_ (10^-4^cm/s) | 1.74 | Predicted |
| ***Distribution -full PBPK model*** | | |
| V_ss_ (L/kg) | 39.13 | Data on file |
| K_p_ scalar | 4.47 | Assigned |
| ***Elimination-in vivo clearance*** | | |
| CL_iv_ (L/h) | 100 | Data on file |
| Percentage CL_H_ by CYP3A4 | 100 | Assigned |
| CL_R_ (L/h) | 19.3 | [Data](#z337ya) on file |
| ***Interaction-CYP3A4 inhibition*** | | |
| Competitive inhibition | | |
| K_i_ (µM) | 4.4 | Data on file |
| f_u, mic_ | 1 | Simcyp default value |
| Time-dependent inhibition | | |
| K_app_ (µM) | 9.66 | Data on file |
| K_inact_ (1/h) | 2.6 | Data on file |
| f_u, mic_ | 1 | Simcyp default value |

MW- molecular weight; B/P, blood to plasma partition ratio; fu,plasma- fraction unbound in plasma; Fafraction absorbed; Ka- first order absorption rate constant; fu, gut- unbound fraction of drug in enterocytes; Qgut- a nominal flow in gut model; MDCK- Madin-Darby Canine Kidney; Peff, man – human jejunum effective permeability; Vss- volume of distribution at steady state; Kp- tissue to plasma partition coefficient; CLH- hepatic clearance; CLR- renal clearance; Ki- concentration of inhibitor that supports half maximum inhibition; fu, mic- fraction of unbound drug in the in vitro microsomal incubation; Kapp- concentration of mechanism based inhibitor associated with half maximal inactivation rate; Kinact- inactivation rate.

^a^ reference (Papp 10^-06^ cm/s ) : cimetidine- 1 ; atenolol- 0.1; propranolol- 20.9; verapamil- 11.2; midazolam- 18.8; metoprolol- 24.8.

**Table S2. Input parameters for the enzalutamide PBPK model**

| **Parameter** | **Value** | **Reference** |
| --- | --- | --- |
| MW (g/mol) | 464.44 | [16] |
| LogP | 2.98 | [16] |
| Compound type | Neural | [16] |
| B/P ratio | 0.55 | [16] |
| f_u, plasma_ | 0.024 | [16] |
| ***Absorption-1st order absorption model*** | |  |
| F_a_ | 0.975 | Predicted in Simcyp based on data from [16] |
| K_a_ (1/h) | 1.452 | Predicted in Simcyp based on data from [16] |
| f_u, gut_ | 1 | [16] |
| Q_gut_ (L/h) | 12.17 | [16] |
| Caco-2 (10^-6^ cm/s) | 31 | [16] |
| Permeability predication scalar | 1 | [16] |
| P_eff, man_ (10^-4^cm/s) | 3.324 | Predicted in Simcyp based on data from [16] |
| ***Distribution -full PBPK model*** | |  |
| V_ss_ (L/kg) | 1.119 | Predicted in Simcyp using Method 1 based on data from [16] |
| K_p_ scalar | 1 | [16] |
| ***In vivo clearance*** | | |
| CL_po_ (L/h) | 0.54 | [16] |
| ***Interaction-CYP3A4 inhibition and induction*** | | |
| Competitive inhibition | | |
| K_i_ (µM) | 42 | [16] |
| f_u, mic_ | 1 | [16] |
| Induction | | |
| Ind C_50_ (µM) | 2.5 | [16] |
| Ind max | 8 | [16] |

**Table S3. Input parameters for the palbociclib PBPK model**

| **Parameter** | **Value** | **Reference** |
| --- | --- | --- |
| MW (g/mol) | 447.54 | [18] |
| LogP | 0.99 | [18] |
| pKa | 4.1 and 7.3 | [18] |
| B/P ratio | 1.63 | [18] |
| f_u, plasma_ | 0.147 | [18] |
| ***Absorption-1st order absorption model*** | |  |
| F_a_ | 0.85 | [18] |
| K_a_ (1/h) | 0.12 | [18] |
| Lag time (h) | 1.66 | [18] |
| f_u, gut_ | 0.73 | [18] |
| Q_gut_ (L/h) | 11.28 | [18] |
| ***Distribution -full PBPK model*** | |  |
| V_ss_ (L/kg) | 14.558 | [18] |
| K_p_ scalar | 1.22 | Assigned in Simcyp using Method 2 to get reported V_ss_ |
| ***Elimination*** | | |
| CL_int_,_CYP3A4_ (μL/min/pmol) | 0.44 | [18] |
| CL_int,others_ (μL/min/mg protein) | 37.65 | [18] |
| CL_R_ (L/h) | 6.6 | [18] |
| ***Interaction-CYP3A4*** | | |
| Time-dependent inhibition | | |
| K_app_ (µM) | 10 | [18] |
| K_inact_ (1/h) | 2.16 | [18] |

**Table S4. Observed and predicted AUCs of ipatasertib following single and multiple oral doses at the dose range of 25-800mg**

|  | **Day 1** |  |  | **Steady state** |  |  |
| --- | --- | --- | --- | --- | --- | --- |
| **Dose (mg)** | **Observed AUC_0-∞_ (ng*hr/ml)** | **Predicted AUC_0-∞_ (ng*hr/ml)** | **P/O** | **Observed AUC_0-24_ (ng*hr/ml)** | **Predicted AUC_0-24_ (ng*hr/ml)** | **P/O** |
| 25 (n=3) | 34.0 (107%) | 58.2 (50%) | 1.71 | 88.3 (77.8%) | 64.6 (54%) | 0.73 |
| 50 (n=3) | 84.6 (50.1%) | 130.6 (51%) | 1.54 | 171 (11%) | 147 (55%) | 0.86 |
| 100 (n=3) | 365 (21.5%) | 308.7 (51%) | 0.85 | 568; 239 | 357.2 (57%) | 0.89 |
| 200 (n=3) | 999 (21.2%) | 776.5 (50%) | 0.78 | 1770 (14.6%) | 955.2 (58%) | 0.54 |
| 400 (n=3) | 2230 (14.1%) | 2044.9 (46%) | 0.92 | 3210 (22.3%) | 2860.7 (58%) | 0.89 |
| 600 (n=7) | 3460 (36.4%) | 3603.4 (43%) | 1.04 | 4450 (43.8%) | 5705.8 (56%) | 1.28 |
| 800 (n=7) | 4100 (68.6%) | 5360.9 (42%) | 1.31 | 6670 (66.1%) | 9403.5 (52%) | 1.41 |

AUC_0- ∞_ and AUC_0-24_ are reported as geometric mean (CV%); P/O- predicted/observed values. At steady state, only two subjects dosed with 100 mg ipatasertib had evaluable observed AUCs.

**Table S5. The predicted and observed pharmacokinetic parameters of enzalutamide and palbociclib**

|  | **Observed AUC_ss_ (µg*hr/ml)** | **Predicted AUC_ss_ (µg*hr/ml)** | **P/O** | **Observed C_max_ (µg/ml)** | **Predicted C_max_ (µg/ml)** | **P/O** |
| --- | --- | --- | --- | --- | --- | --- |
| Enzalutamide | 341 (25%) | 320 (28%) | 0.94 | 17 (22%) | 15 (25%) | 0.88 |
|  | **Observed AUC_ss_ (ng*hr/ml)** | **Predicted AUC_ss_ (ng*hr/ml)** | **P/O** | **Observed C_max_ (ng/ml)** | **Predicted C_max_ (ng/ml)** | **P/O** |
| Palbociclib | 2565 (25%) | 2612 (53%) | 1.02 | 123 (22%) | 134 (48%) | 1.09 |

AUC_ss_ and C_max_  are reported as geometric mean (CV%); P/O- predicted/observed values.

**Table S6.** **Predicted pharmacokinetic parameters of ipatasertib at steady state following 400mg ipatasertib alone and 200 mg ipatasertib in the presence of moderate CYP3A4 inhibitors**

|  | **Ipatasertib**  **AUC_ss_**  **(ng*hr/ml)** | **Ipatasertib**  **C_max_ (ng/ml)** | **Ipatasertib**  **T_max_**  **(hr)** |
| --- | --- | --- | --- |
| Ipatasertib 400 mg | 2936  (60%) | 289  (39%) | 2.02  (1.01-3.05) |
| Ipatasertib 200 mg with erythromycin | 2970  (45%) | 210  (35%) | 2.26  (1.42-3.16) |
| Ipatasertib 200 mg with diltiazem | 2292  (54%) | 182  (39%) | 2.16  (1.16-3.05) |

AUC_ss_ and C_max_ are reported as geometric mean (CV%); T_max_ is reported as median (range: minimum-maximum).
