# Supplementary material for: Assessment of cytochrome P450 3A4-mediated drug–drug interactions for ipatasertib using a fit-for-purpose physiologically based pharmacokinetic model
Source: Cancer Chemother Pharmacol. 2022 Apr 15;89(5):707–20. doi: 10.1007/s00280-022-04434-2 (PMC9054915; doi:10.1007/s00280-022-04434-2)
Supplement: Supplementary file 2 — Supplementary file2 (PDF 270 KB) [file 280_2022_4434_MOESM2_ESM.pdf]

Figure S1. Simulated and observed plasma concentration-time profiles of ipatasertib following (a) a single IV dose of 0.08 mg, (b) a single oral dose and (c) multiple oral doses

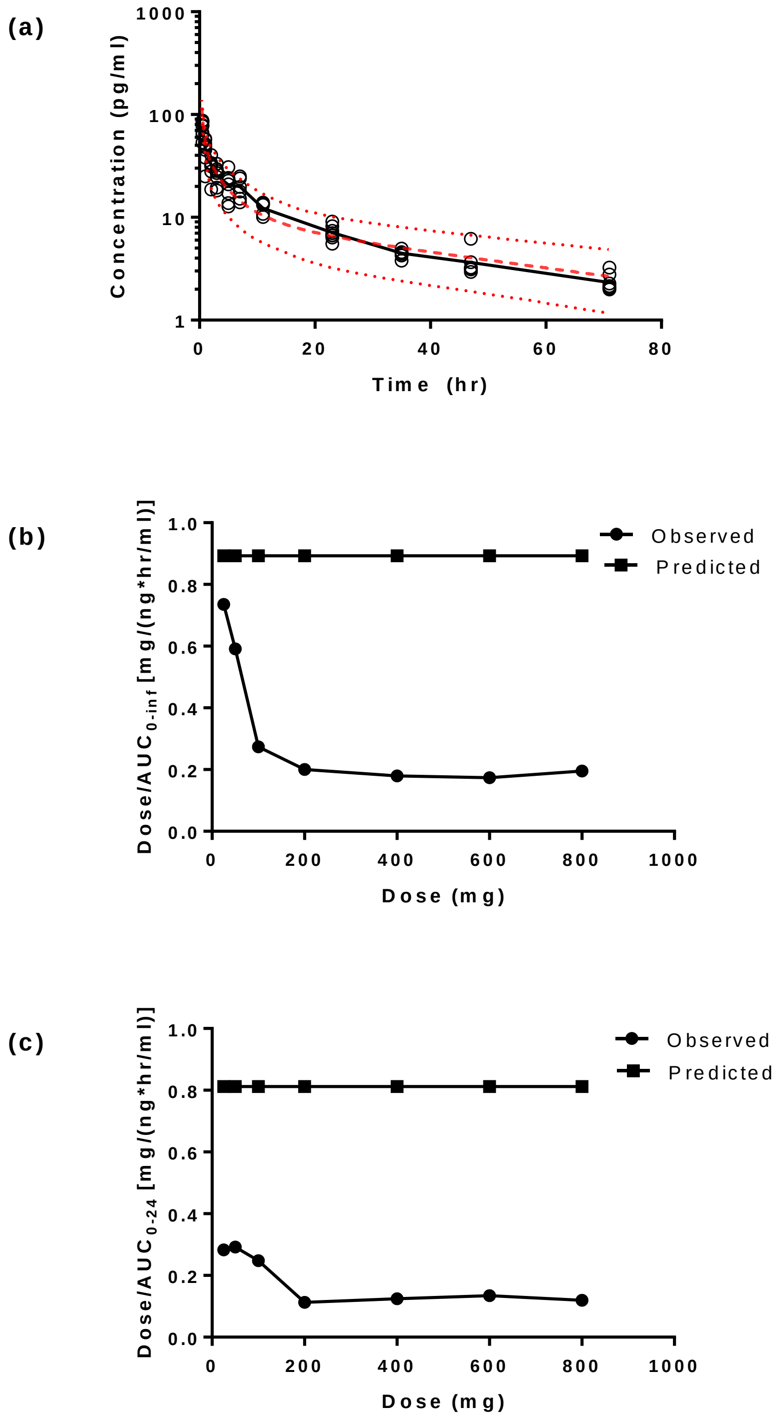

Solid line- observed mean plasma concentrations; circles- observed individual plasma concentrations; dashed lines- predicted mean, 95th and 5th percentile.
