# Supplementary material for: Assessment of cytochrome P450 3A4-mediated drug–drug interactions for ipatasertib using a fit-for-purpose physiologically based pharmacokinetic model
Source: Cancer Chemother Pharmacol. 2022 Apr 15;89(5):707–20. doi: 10.1007/s00280-022-04434-2 (PMC9054915; doi:10.1007/s00280-022-04434-2)
Supplement: Supplementary file 3 — Supplementary file3 (PDF 202 KB) [file 280_2022_4434_MOESM3_ESM.pdf]

Figure S2. Sensitivity analyses of CYP3A4 (a) competitive inhibition and (b) time-dependent inhibition for midazolam AUC changes after a 2mg single oral dose in the presence of 600 mg ipatasertib QD

(a)

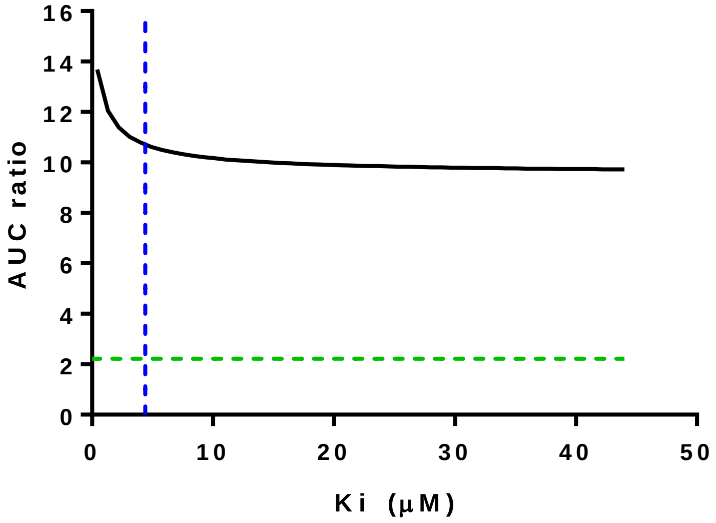

(b)

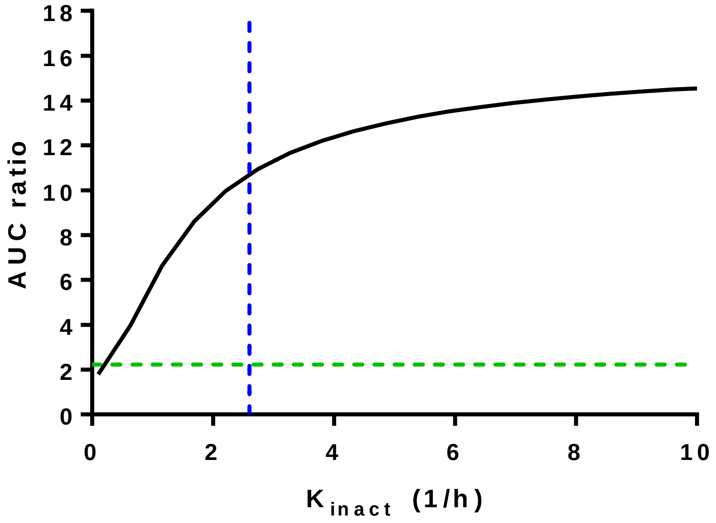

Green dashed line- observed midazolam AUC ratio of 2.22; blue dashed line- *in vitro* estimated K_i of 4.4 μM and K_{inact} of 2.6 1/h. Note: Shown here is the sensitivity analysis of the model with K_m of 0.195 μM. Same conclusions are drawn from the other two models with Km of 1.95 μM and 19.47 μM.
