# Supplementary material for: Assessment of cytochrome P450 3A4-mediated drug–drug interactions for ipatasertib using a fit-for-purpose physiologically based pharmacokinetic model
Source: Cancer Chemother Pharmacol. 2022 Apr 15;89(5):707–20. doi: 10.1007/s00280-022-04434-2 (PMC9054915; doi:10.1007/s00280-022-04434-2)
Supplement: Supplementary file 4 — Supplementary file4 (PDF 563 KB) [file 280_2022_4434_MOESM4_ESM.pdf]

Figure S3. Simulated and observed plasma concentration-time profiles of (a) 200 mg QD itraconazole after the fourth dose, (b) ipatasertib after a single IV dose of 0.08 mg and (c) ipatasertib after a single oral dose of 200 mg

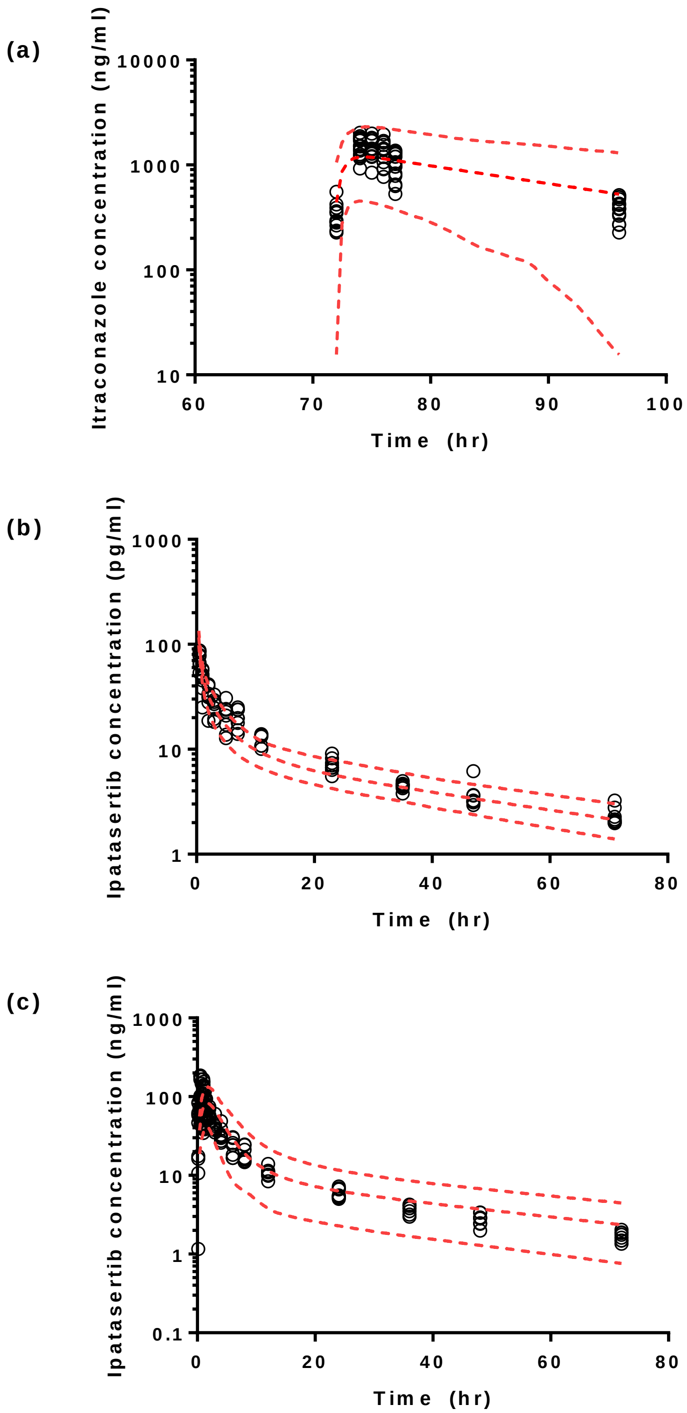

Circles - observed individual plasma concentrations; dashed line - predicted mean plasma concentrations, 95th and 5th percentile.
